# Assessment of a LPG hybrid solar dryer assisted with smart air circulation system for drying basil leaves

**DOI:** 10.1038/s41598-024-74751-4

**Published:** 2024-10-13

**Authors:** El-Sayed Gomaa Khater, Adel Hamed Bahnasawy, Awad Ali Tayoush Oraiath, Sadeq K. Alhag, Laila A. Al-Shuraym, Moustapha Eid Moustapha, Abdallah Elshawadfy Elwakeel, Ahmed Elbeltagi, Ali Salem, Khaled A. Metwally, Mohamed A. I. Abdalla, Mahmoud M. Hussein, Mohamed Anwer Abdeen

**Affiliations:** 1 Agricultural and Biosystems Engineering Department, Faculty of Agriculture, Benha University, Moshtohor, P.O. Box 13736, Toukh, Kalubia Egypt; 2 Department of Agricultural Engineering, Faculty of Agriculture, Omar Al Mukhtar University, 991 Al Bayda, Libya; 3 Biology Department, College of Science and Arts, King Khalid University, Muhayl Asser, 61913 Saudi Arabia; 4 Biology Department, Faculty of Science, Princess Nourah Bint Abdulrahman University, Riyadh, Saudi Arabia; 5 Department of Chemistry, College of Science and Humanities, Prince Sattam bin Abdulaziz University, Al-Kharj, 11942 Saudi Arabia; 6 Agricultural Engineering Department, Faculty of Agriculture and Natural Resources, Aswan University, Aswan, Egypt; 7 Agricultural Engineering Department, Faculty of Agriculture, Mansoura University, Mansoura, 35516 Egypt; 8 Civil Engineering Department, Faculty of Engineering, Minia University, Minya, Egypt 61111; 9 Structural Diagnostics and Analysis Research Group, Faculty of Engineering and Information Technology, University of Pecs, Pecs, Hungary; 10 Soil and Water Sciences Department, Faculty of Technology and Development, Zagazig University, Zagazig, 44519 Egypt; 11 Department of Soil and Water Conservation, Desert Research Center, Cairo, 11753 Egypt; 12 Department of Communications Technology Engineering, Technical College, Imam Ja’afar Al-Sadiq University, Baghdad, 10053 Iraq; 13 College of Engineering, South China Agricultural University, Guangzhou, 510642 China; 14 Agricultural Engineering Department, College of Agriculture, Zagazig University, Zagazig, 44519 Egypt

**Keywords:** Automation, Medicinal plants, Mathematical modeling, Internet of things (IoT), Food quality, Postharvest technology, Energy harvesting, Environmental impact

## Abstract

The fluctuation of solar radiation throughout the day presents a significant obstacle to the widespread adoption of solar dryers for the dehydration of agricultural products, particularly those that are sensitive to high temperatures, such as basil leaf drying during the winter season. Consequently, this recent study sought to address the limitations of solar-powered dryers by implementing a hybrid drying system that harnesses both solar energy and liquid petroleum gas (LPG). Furthermore, an innovative automatic electronic unit was integrated to facilitate the circulation of air between the drying chamber and the ambient environment. Considering the solar radiation status in Egypt, an LPG hybrid solar dryer has been developed to be suitable for both sunny and cloudy weather conditions. This hybrid solar dryer (HSD) uses indirect forced convection and a controlled auxiliary heating system (LPG) to regulate both temperature and relative humidity, resulting in increased drying rates, reduced energy consumption, and the production of high-quality dried products. The HSD was tested and evaluated for drying basil leaves at three different temperatures of50, 55, and 60 °C and three air changing rates of 70, 80, and 90%, during both summer and winter sessions. The obtained results showed that drying basil at a temperature of 60 °C and an air changing rate of 90% led to a decrease in the drying time by about 35.71% and 35.56% in summer and winter, respectively, where summer drying took 135–210 min and winter drying took 145–225 min to reach equilibrium moisture content (MC). Additionally, the effective moisture diffusivity ranged from 5.25 to 9.06 × 10^− 9^ m^2^/s, where higher values of effective moisture diffusivity (EMD) were increased with increasing both drying temperatures and air change rates. Furthermore, the activation energy decreased from 16.557 to 25.182 kJ/mol to 1.945–15.366 kJ/mol for the winter and summer sessions, respectively. On the other hand, the analysis of thin-layer kinetic showed that the Modified Midilli II model has a higher coefficient of determination R^2^, the lowest χ^2^, and the lowest root mean square error (RMSE) compared to the other models of both winter and summer sessions. Finally, the LPG hybrid solar dryer can be used for drying a wide range of agricultural products, and it is more efficient for drying medicinal plants. This innovative dryer utilizes a combination of LPG and solar energy, making it efficient and environmentally friendly.

## Introduction

Medicinal plants have been considered a primary source of traditional medicine throughout history.

Archaeological evidence has revealed historical documentation of herbs from ancient civilizations such as the Sumerian civilization, which recorded numerous therapeutic plants on clay tablets, and ancient Egypt, where over 850 plant-based medicines were meticulously cataloged^1^. Basil (*Ocimum baliscum*) is one of the most important medicinal plants in the *Lamiaceae* family. It is an annual-growing herbaceous plant, with purple flowers and a length of 20–60 cm. Basil originated in Iran and India, and it is cultivated in Mediterranean countries with high-temperate and dry climates^2,3^. Besides its significant commercial value, people use basil as a spice in food preparation, a fresh vegetable, a medicinal plant, an antimicrobial, an antiviral, and an antioxidant^4,5^.

Basil can be taken either in its fresh or dried form. While, fresh basil have a short shelf life and quickly lose their color, aroma, and texture, so there has been an increase in the use of dry products^6^. Globally, food companies employ various preservation techniques to mitigate the growth of harmful germs, maintain the nutritional content of food, minimize agricultural waste, and lower production costs^7^.

Open sun drying (OSD) is classified as a direct method, when the product is heated directly by sunlight radiation, which involves exposing agricultural products, including medicinal herbs, to direct sunlight, is the most cost-effective and uncomplicated technique of drying. Exposure to direct sunlight causes herbs to lose their fragrance and change color, reducing their attractiveness to customers. Furthermore, there is a possibility of bug infestations, bird droppings, rodent attacks, and other similar incidents occurring^8^. Nevertheless, the exorbitant expenses and substantial energy demands associated with food processing serve as constraints for the majority of conservation methods.

The food industry’s energy use for storage, processing, and distribution accounts for approximately 43% of its total energy usage^9^. Among these percentages, the process of drying stands out as the operation that requires the most energy. This is because removing water from the food matrix incurs a significant energy cost^10^. A number of academics conducted an investigation into the process of drying basil. Utilizing various apparatus, such as microwave dryers^11^, vacuum microwave dryers^12^, freeze dryers^13^, sun drying^14^, shade drying^15^, and solar dryers (SD)^16^, SD are both ecologically sustainable and devoid of any pollution. Their designs are contingent upon the capacity, inclination, and accessibility of materials and resources^16–19^. The indirect forced convection solar dryer exhibits superior speed and efficiency compared to alternative variations^20^. It is also sequentially administered to dehydrated medicinal plants such as peppermint and thyme^21^. The desiccated product is evenly spread on trays within a thermally insulated drying room (DR), ensuring that it is not directly exposed to solar radiation. A solar air heater (SAH) is required to warm the air used for drying. The SAH utilized for drying purposes is enhanced through the incorporation of fins^22–24^ and V-corrugated absorber plates^25,26^. However, maintaining a consistent temperature inside the DR is unattainable due to the fluctuation in solar radiation intensity reaching the solar collector during the day.

Consequently, researchers globally are actively seeking alternative solutions to address this challenge. Where, the HSD has the capability to produce a higher temperature in the surrounding air compared to ordinary solar dryers^27^. Raising the temperature of the room air accelerates the process of removing moisture removed from the crop surface. Consequently, this allows for a greater quantity of crops to be dried in the dryer in a shorter period of time. In addition to its primary source of thermal energy, the HSD also utilizes solar thermal energy as an auxiliary source^28^. Therefore, following sunset, the dryer continues to function by utilizing an alternative energy source such as, biomass^29^, LPG^30,31^, solar collectors^32^, etc., or energy storage materials^33^. Furthermore, the design of HSD specifically targets the drying of crops with high moisture content (MC). This is necessary since such crops need to be dried quickly in order to prevent them from emitting unpleasant odors and degrading^34^.

The Thin-Layer (ThL) Kinetic describes the drying process. Where ThL Kinetics has been utilized to calculate the drying times of numerous items and generate curves. ThL drying is the process of extracting moisture from a substance after exposing it to a constant relative humidity^35^. Experimental circumstances in research employing this strategy were shown to be correlated with drying parameters^36,37^. Researchers have utilized various ThL models to simulate the drying kinetics of basil^14,37,38^, However, their primary emphasis was on examining the impact of a limited number of drying apparatuses on the drying kinetics of basil. Based on our current understanding, there is insufficient data available regarding the drying kinetics of purple basil leaves and the energy efficiency of the systems used to dry them.

The fluctuation of solar radiation over the day poses a considerable challenge to the extensive implementation of solar dryers for dehydrating agricultural goods, especially those vulnerable to elevated temperatures, such as leaf drying in winter and cloudy days. This recent study aimed to overcome the limitations of solar-powered dryers by developing a hybrid drying system that utilizes both solar energy and liquefied petroleum gas (LPG). Additionally, to enhance dryer efficiency and reduce energy wastage, a novel electronic unit was incorporated to promote air circulation between the drying chamber and the surrounding environment. In current studies, the impact of the drying air temperature, air changing rate, and drying session on changes in the drying parameters, thin-layer kinetic, diffusivity, and activation energy of the basil leaves drying process have not been investigated; hence, optimizing the drying process of basil leaves, the current paper focused on the development of an LPG hybrid solar dryer integrated with smart control system and study the influence of the drying air temperature, air changing rate, and drying session on changes in the weight loss, drying rate, moisture ratio (MR), drying coefficient, determination coefficient, effective moisture diffusivity (EMD), activation energy, drying kinetics, to improve the drying process of basil leaves.

## Materials and methods

### Biological material to be dried

**Fig. 1:**
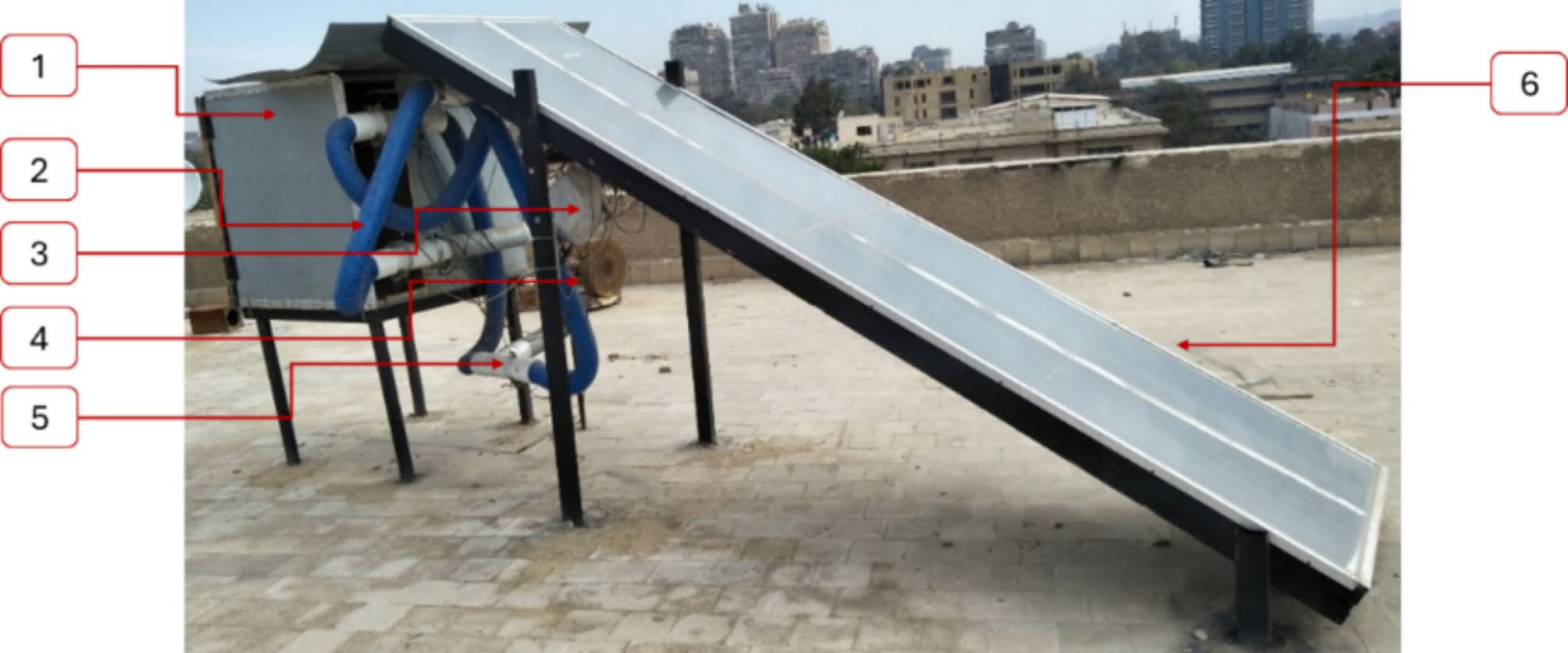
Main components of the HSD. Whereas (1) Drying room; (2) air pipes; (3) LPG burner; (4) blower; (5) air gates; (6) solar collector^39^.

The fresh basil (*Ocimum basilicum L.*) was harvested from the experimental research station at Benha University. Initially, the moisture content (MC) ranged from 77 to 87% (w.b.). After harvesting, we removed the basil stems and washed the leaves to remove any impurities. After using blotting paper to remove excess moisture, we meticulously weighed 2 kg of basil. Subsequently, the basil was evenly spread onto drying trays within the designated DR with a 2 cm layer thickness The HSD was designed, constructed, and evaluated at the Agricultural and Bio-Systems Engineering Department, Faculty of Agriculture, Moshtohor, Benha University, Egypt. Field tests for the HSD is conducted during two seasons of 2023: winter (January and February) and summer (July to September). Figure 1 shows the HSD’s main components.2.2. Description of the developed solar dryer.

The HSD was developed and used to achieve the current study’s goals. It was integrated with a flat plate solar collector (FPSC) and assisted by a LPG burner to adjust and raise the air temperature inside the DR.

**Fig. 2:**
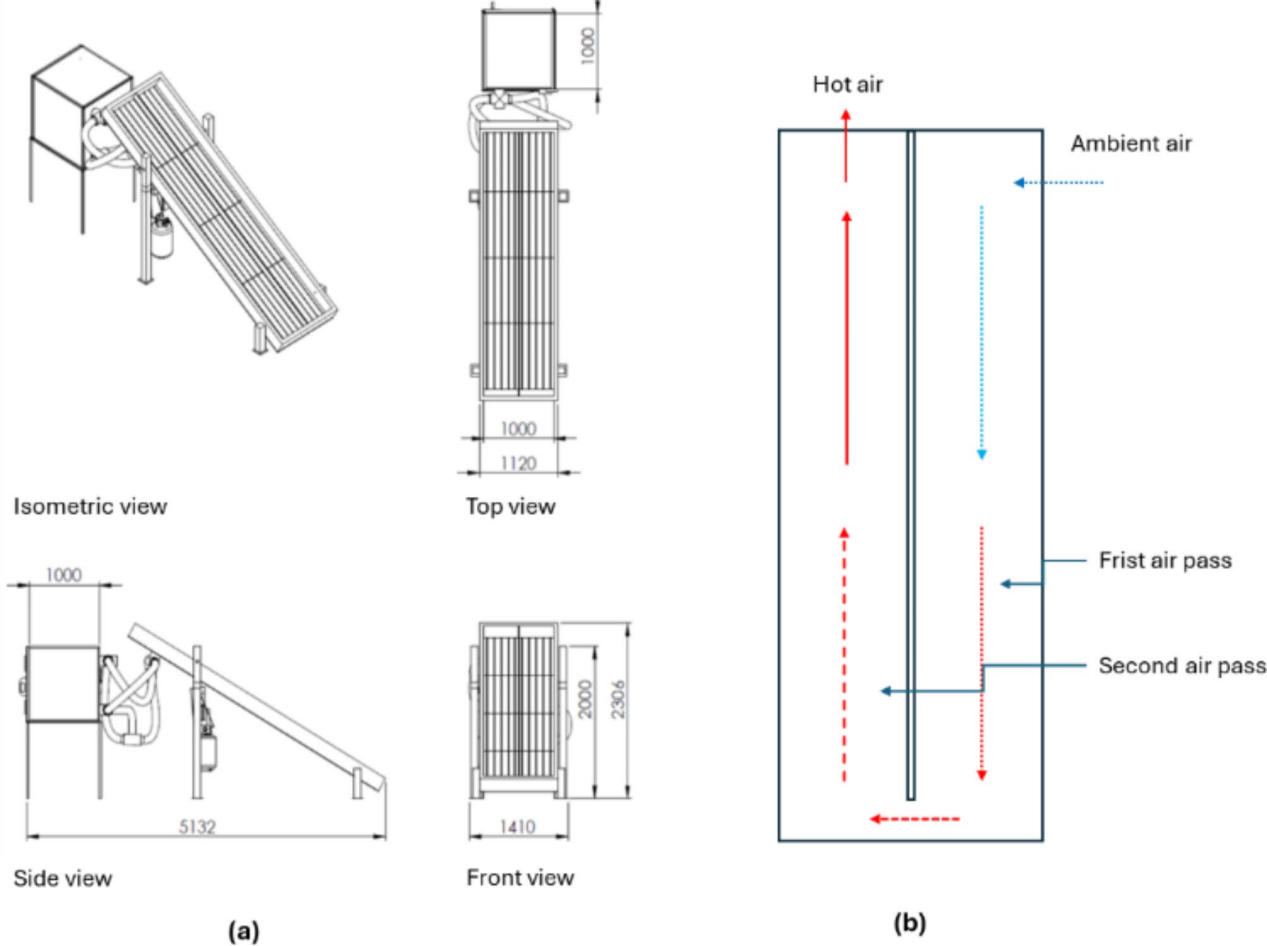
The schematic drawing of the HSD, whereas, (**a**) different detailed and isometric views of the HSD, all dimensions in mm; (**b**) air circulation passes inside the FPSC^39^.

Figure 2a depicts various detailed and isometric views of the HSD, showcasing the FPSC’s main dimensions of 4 m in length and 1 m in width, insulated from three sides with 5 cm thermal wood. The absorber plate was manufactured from an aluminum corrugated sheet and then painted black. Finally, the FPSC was covered with a 4 mm glass-tempered sheet. To increase the heating period, the inlet air was divided into two symmetric passes before entering the DR, as shown in Fig. 2b.

**Fig. 3:**
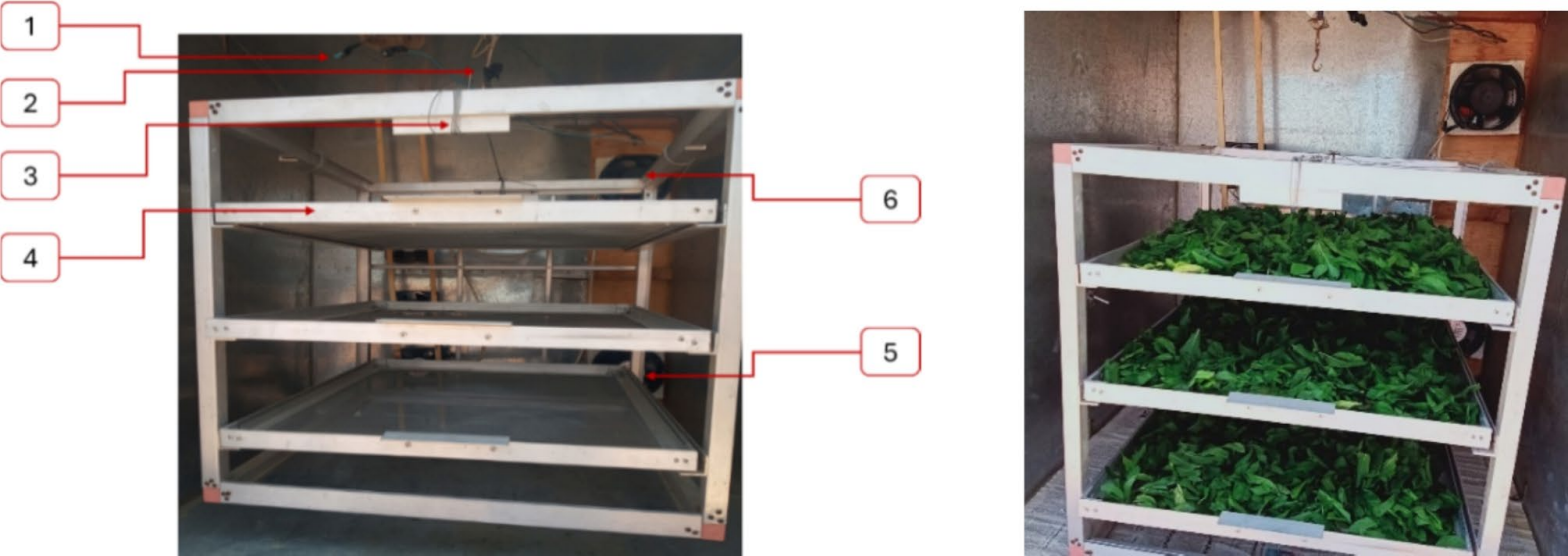
Internal components of the DR. Whereas (1) two DHT-22 (temperature and humidity sensor); (2) hinged point; (3) load cell; (4) drying trays; (5) Outlet air blower; (6) Inlet air blower.

The HSD is accompanied by a distinct DR, as depicted in Fig. 2a. The DR possesses dimensions of 1 m in width, 1 m in height, and 1 m in depth, resulting in a total volume of 1 m^3^. Figure 3 illustrates the configuration of the DR, featuring three drying trays affixed to a lightweight aluminum frame. A load cell integrates with the aluminum frame, pivotally attached at the midpoint of the DR’s upper section. The drying system utilized two air blowers (Model C.C.P. Parma—6.6 m^3^/h—2800 rpm–150 W, 220 V 50 Hz, Italy) were used to recirculate the hot air inside the drying system, (Fig. 3). Also, the HSD assisted with a LPG burner to raise the temperature of the outgoing air from the FPSC before entering the DR and the temperature of the circulated air. The burner features switches equipped with a sparking mechanism that initiates the combustion of LPG supplied from the LPG bottle.

**Fig. 4:**
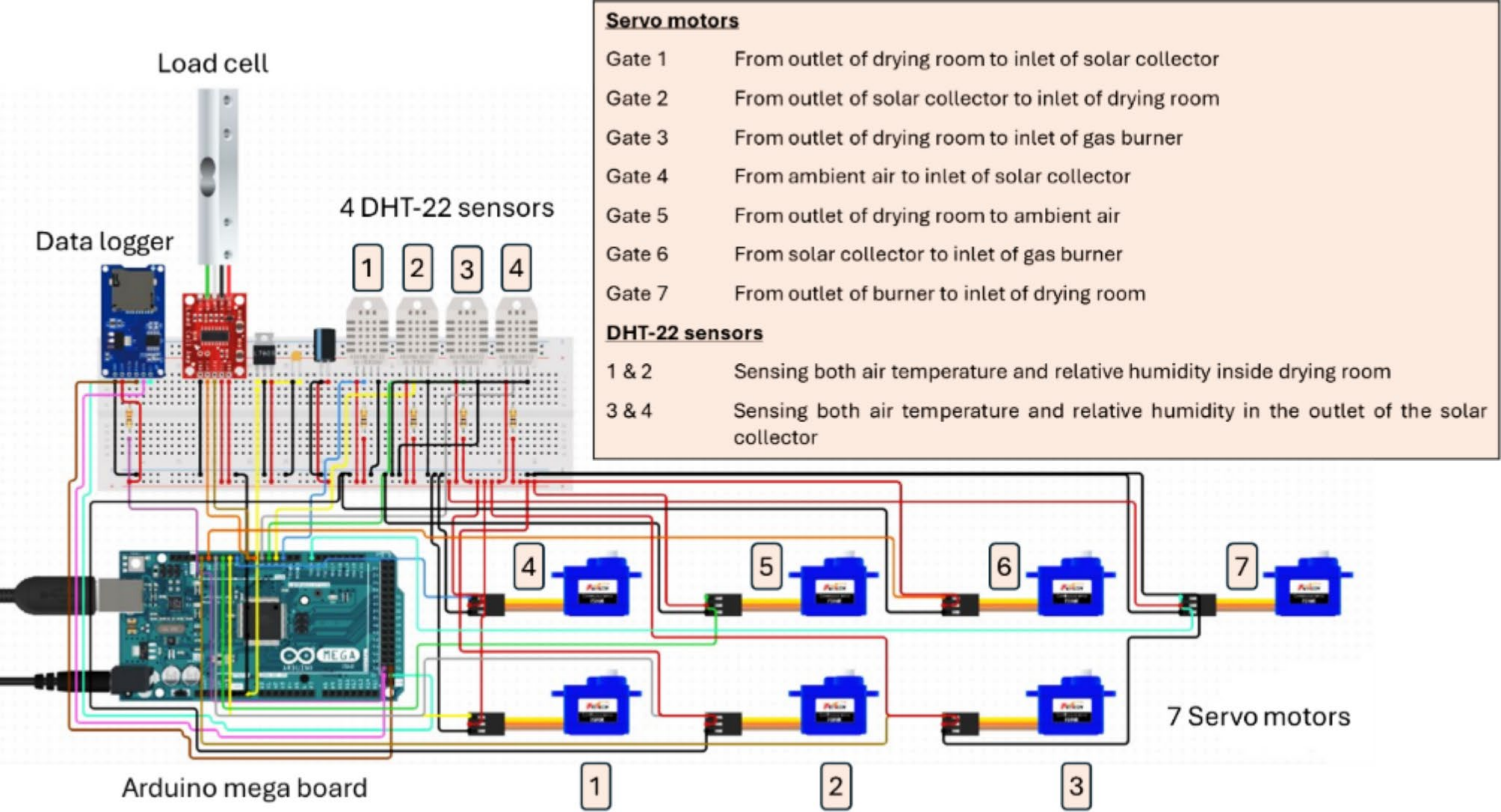
Different components of the measuring, data storage, and control circuit.

**Fig. 5:**
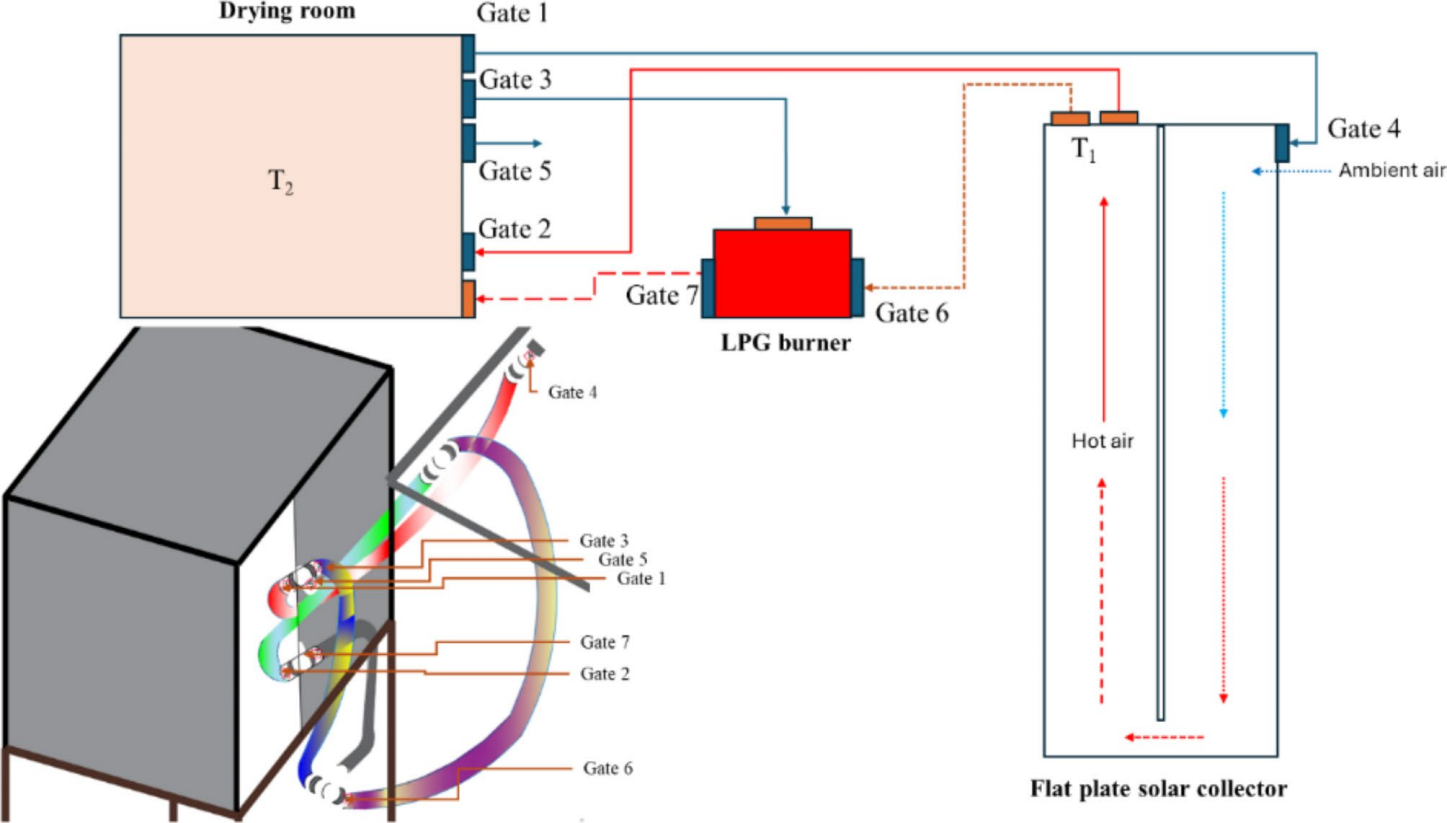
The position of different gates to regulate the air circulation between the FPSC, the LPG burner, and the DR.

**Fig. 6:**
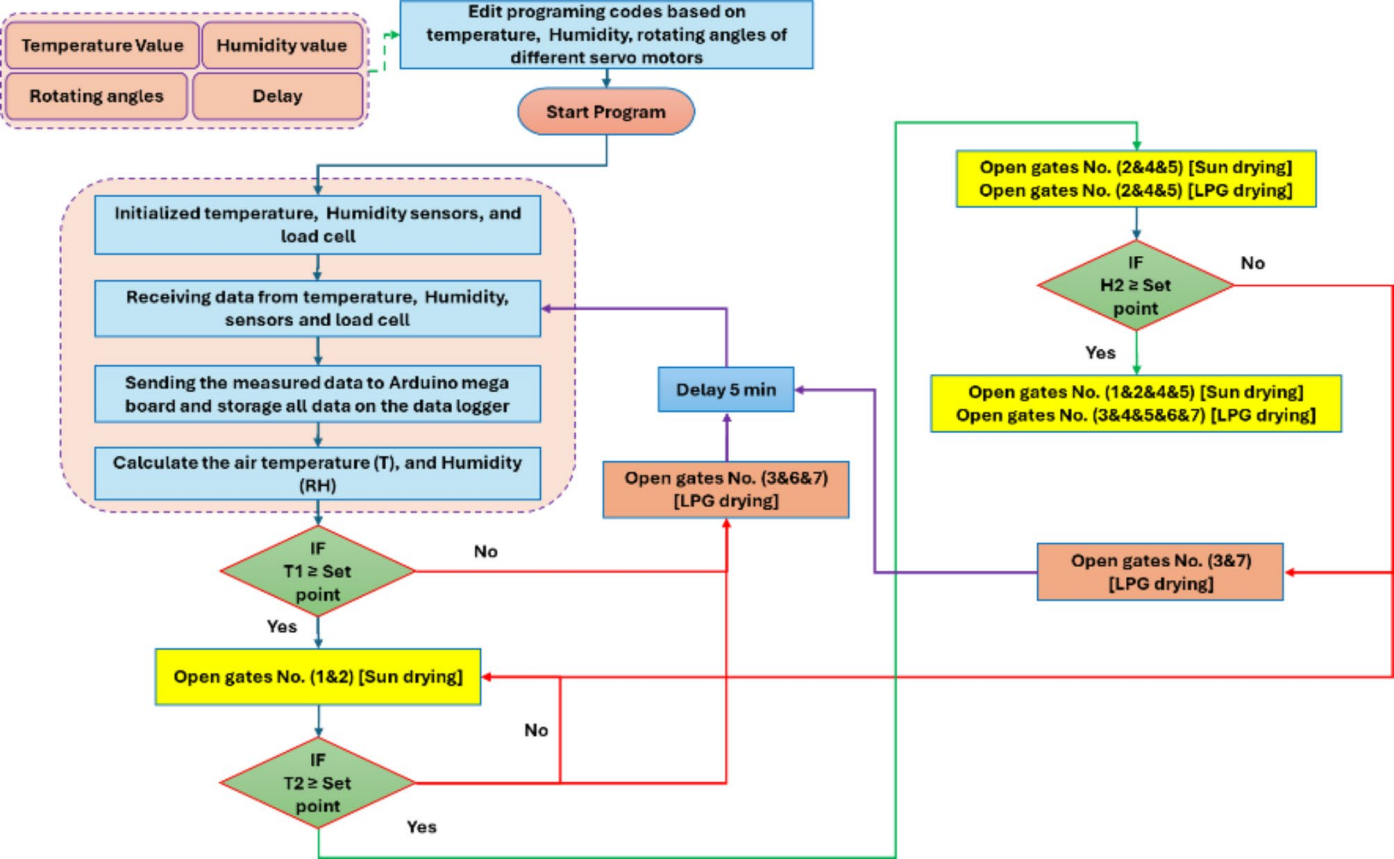
The operating principles of the air circulation system inside the DR, the LPG burner, and the FPSC.

The HSD was equipped with a specifically designed control circuit. This circuit was developed to monitor the air temperature and relative humidity inside the DR and at the outlet of the FPSC. The design also included measuring the initial and interval weight of the basil and storing the temperature, relative humidity, and basil weight on a data logger. Furthermore, the control circuit was engineered to regulate the opening and closing processes of the seven air circulation gates between the FPSC, DR, LPG burner, and the outlet of the FPSC. The measuring, data storage, and control circuit (Fig. 4) comprised several precision electronic components, including an Arduino Mega board, seven servo motors, four DHT-22 sensors, a load cell, and a data logger. Figure 4 shows the measuring, data storage, and control circuit components. Figure 5 illustrates the position of different gates to regulate the air circulation between the FPSC, the LPG burner, and the DR. Figure 6 presents the operating principles of the air circulation system inside the DR, the LPG burner, and the FPSC.

### Design of the experiment

The drying process of the basil samples will be done using the HSD developed in the current study to investigate the effects of the independent variables on the dependent variables during the experiments, where three levels of the drying air temperatures (50, 55, and 60 ◦C) and three levels of the air changing rates (70, 80, and 90%) were used on the responses of the weight loss, drying rate, MR, drying coefficient, determination coefficient, EMD, activation energy, and drying kinetics to improve the drying process of basil.

### Evaluation of an LPG hybrid solar dryer

####  Drying parameters

##### Moisture content (MC)

An electric oven estimated the initial MC of the basil leaves to dry the basil leaves until equilibrium MC at 55 °C^40^. Then, the basil leaf samples’ initial MC (w.b.) was calculated using Eq. (1), as mentioned in Refs.^41–45^

1 $$\:\text{M}\text{C}=\left[\frac{{\text{W}}_{\text{w}}-\:{\text{W}}_{\text{d}}}{{\text{W}}_{\text{w}}}\right]\times\:100\:$$

Where, $\:{\text{W}}_{\text{w}}\:\text{a}\text{n}\text{d}\:{\text{W}}_{\text{d}}$ is the wet and dry weight of basil leaf samples.

##### Weight loss (WL)

The weight loss of the different basil leaf samples was calculated by subtracting the basil leaf samples’ interval weight, using Eq. 2, as reported in Refs^19,46^.

2 $$\:\text{W}\text{L}={\text{W}}_{\text{t}}-\:{\text{W}}_{\text{t}+1}\:\:$$

##### Drying rate

The drying rate was calculated using Eq. 3, as described in Refs^47–49^ where this equation is the most widely used for estimation the drying rate because of its high accuracy.

3 $$\:\text{D}\text{r}\text{y}\text{i}\text{n}\text{g}\:\text{r}\text{a}\text{t}\text{e}\:\:=\:\frac{{\text{M}\text{C}}_{(\text{t}+\text{d}\text{t})}-{\text{M}\text{C}}_{\text{t}}}{{\text{d}}_{\text{t}}}\:$$

##### Moisture ratio (MR)

As recommended by Rabha et al.^50^, Eq. 4 was used for the calculation of the MR during the drying process of basil leaves.

4 $$\:\text{M}\text{R}=\frac{{\text{M}\text{C}}_{\text{t}}}{{\text{M}\text{C}}_{0}}\:\:\:\:$$

where, $\:{\text{M}\text{C}}_{\text{t}}\:$is the MC at any time, and $\:{\text{M}\text{C}}_{0}$ is the initial MC.

##### Drying coefficient (k)

Drying constant discusses heat and mass transfer methods and examines how process factors affect moisture removal. It is the most important constant in the mathematical model of any dehydration operation, which estimates drying time and the behavior of all operational elements that affect dryer design and optimization. The drying constant is determined by experimental investigations of the moisture content reduction of materials over time under varying drying conditions. The assessment of material moisture content over time under consistent drying air conditions is referred to as the drying curve. Where drying coefficient (k) was calculated using Eq. 5, as noted by^19,51,52^.

5 $$\:\text{M}\text{R}=\text{A}\text{exp}(-\text{k}\times\:\text{t})$$

##### Effective moisture diffusivity (EMD) and activation energy (E_a_)

Fick’s second law of diffusion is a widely employed principle for explaining the phenomenon of moisture diffusion, especially when measuring EMD^53^, as follows:

6 $$\:\frac{\partial\:\text{M}\text{C}}{\partial\:\text{t}}={\text{D}}_{\text{e}\text{f}\text{f}}\times\:{\nabla\:}^{2}\text{M}\text{C}$$

The diffusion analysis assumes that the original MC is the same, that the main resistance is internal moisture movement, that there is almost no resistance to heat and mass transfer from the outside, and that the effective diffusion coefficient stays the same^54^. A series-type equation gives the average concentration of a diffusing substance:

7 $$\:\text{M}\text{R}=\:\frac{8}{{{\uppi\:}}^{2}}\times\:\sum\limits_{\text{n}=1}^{{\infty}}\frac{1}{{\text{n}}^{2}}\text{e}\text{x}\text{p}\left(\frac{{-{\uppi\:}}^{2}\times\:{\text{D}}_{\text{e}\text{f}\text{f}}\times\:\text{t}}{4{\text{L}}^{2}}\right)$$

where, n is the term number; t is the time in s; $\:\text{L}$ is the slab thickness.

Equation 8 can be simplified by taking the first term of Eq. 7.

8 $$\:\text{M}\text{R}=\:\frac{8}{{{\uppi\:}}^{2}}\times\:\text{A}\:\text{e}\text{x}\text{p}\left(\frac{-{{\uppi\:}}^{2}\times\:{\text{D}}_{\text{e}\text{f}\text{f}}\times\:\text{t}}{4{\text{L}}^{2}}\right)\:$$

Also, Eq. 9 has been obtained mathematically from Eq. 8.

9 $$\:\text{ln}\left(\text{M}\text{R}\right)=\text{ln}\left(\frac{8}{{{\uppi\:}}^{2}}\right)-\:\left(\frac{{{\uppi\:}}^{2}\times\:{\text{D}}_{\text{e}\text{f}\text{f}}\times\:\text{t}}{4{\text{L}}^{2}}\right)$$

Finding the diffusion coefficient involves plotting experimental drying data in terms of ln (MR) versus time, s. The activation energy was also found using the law of Arrhenius, which is done in the same way as the diffusion coefficient^55^.

10 $$\:{\text{D}}_{\text{e}\text{f}\text{f}}=\:{\text{D}}_{0}\text{exp}\left(-\frac{{\text{E}}_{\text{a}}}{\text{R}\text{T}}\right)$$

##### Thin layer (ThL) kinetics

When studying ThL processes, experimental data are usually matched with well-known models that have been shown to accurately describe how leaf products dry (Table 1). These models are more accurate at guessing the drying qualities of different dried goods and need fewer assumptions to work. After that, different ThL drying models were used on the drying graphs (lnMR versus time) (see Table 1). Then, non-linear least squares regression analysis was used to find the parameters of these models. Using Microsoft Excel, it was possible to see how well each model fit the testing data^56,57^. The following criteria were adopted in the selection of the best model: the lowest χ^2^ and RMSE values and the greatest R^2^^58–60^. These parameters were calculated using Eqs. 11–13 according to Refs^50,61–64^.

**Table 1:** Selected mathematical modeling to demonstrate the basil drying process.

No.	Model name	Model equation*	Reference
1	Newton (Lewis)	$\:\text{M}\text{R}=\text{e}\text{x}\text{p}\left(-\text{k}\text{t}\right)$	^65^
2	Page	$\:\text{M}\text{R}=\text{e}\text{x}\text{p}\left(-\text{k}{\text{t}}^{\text{n}}\right)$	^66,67^
3	Simplified Ficks Diffusion	$\:\text{M}\text{R}=\text{a}\text{exp}\left(-\text{c}\left(\frac{\text{t}}{{\text{L}}^{2}}\right)\right)$	^54^
4	Approximation diffusion or (Diffusion Approach)	$\:\text{M}\text{R}=\text{a}\text{exp}\left(-\text{k}\text{t}\right)+\left(1-\text{a}\right)\text{e}\text{x}\text{p}\left(-\text{k}\text{b}\text{t}\right)$	^68,69^
5	Logistics	$\:\text{M}\text{R}=\:\frac{\text{b}}{1+\text{a}\text{exp}\left(\text{k}\text{t}\right)\:}$	^70^
6	Parabolic model	$\:\text{M}\text{R}=\text{a}+\text{b}\text{t}+\text{c}{\text{t}}^{2}$	^71^
7	Combined Two-term and Page	$\:\text{M}\text{R}=\text{a}\:\text{e}\text{x}\text{p}\left(-\text{k}{\text{t}}^{\text{n}}\right)+\text{b}\:\text{e}\text{x}\text{p}\left(-\text{h}{\text{t}}^{\text{n}}\right)$	^72^
8	Modified Henderson and Pabis	$\:\text{M}\text{R}=\text{a}\text{exp}\left(-\text{k}\text{t}\right)+\text{b}\:\text{e}\text{x}\text{p}\left(-\text{g}\text{t}\right)+\text{c}\:\text{e}\text{x}\text{p}\left(-\text{h}\text{t}\right)$	^73^
9	Modified Midilli II	$\:\text{M}\text{R}=\text{a}\:\text{e}\text{x}\text{p}\left(-\text{k}{\text{t}}^{\text{n}}\right)+\text{b}$	^74^
10	Modified Page III	$\:\text{M}\text{R}=\text{k}\:\text{e}\text{x}\text{p}{\left(-\frac{\text{t}}{{\text{d}}^{2}}\right)}^{\text{n}}$	^74^
11	Modified Two Term III	$\:\text{M}\text{R}=\text{a}\:\text{e}\text{x}\text{p}\left(-\text{k}\text{t}\right)+\left(1-\text{a}\right)\:\text{e}\text{x}\text{p}\left(-\text{k}\text{a}\text{t}\right)$	^74^

*k is the drying constant, min^− 1^; t is drying time, min; L is the samples thickness (slab), m; a, b,c, d,g, h, n are the models constants, dimensionless.

11 $$\:{\text{R}}^{2}=1-\frac{\sum\:_{\text{i}=1}^{\text{N}}{{(\text{M}\text{R}}_{\text{p}\text{r}\text{e},\:\text{i}}-{\text{M}\text{R}}_{\text{o}\text{b}\text{s},\:\text{i}})}^{2}}{\sum\:_{\text{i}=1}^{\text{N}}{{(\stackrel{-}{\text{M}}\text{R}}_{\text{p}\text{r}\text{e}}-{\text{M}\text{R}}_{\text{o}\text{b}\text{s},\:\text{i}})}^{2}}\:\:$$

12 $$\:{{\upchi\:}}^{2}=\frac{\sum\:_{\text{i}=1}^{\text{N}}{{(\text{M}\text{R}}_{\text{p}\text{r}\text{e},\:\text{i}}-{\text{M}\text{R}}_{\text{o}\text{b}\text{s},\:\text{i}})}^{2}}{\text{N}-\text{n}}\:$$

13 $$\:\text{R}\text{M}\text{S}\text{E}=\sqrt{\frac{1}{\text{N}}{\sum\:}_{\text{i}=1}^{\text{N}}{{(\text{M}\text{R}}_{\text{p}\text{r}\text{e},\:\text{i}}-{\text{M}\text{R}}_{\text{o}\text{b}\text{s},\:\text{i}})}^{2}}\:$$

where, $\:{\text{M}\text{R}}_{\text{o}\text{b}\text{s},\:\:\text{i}}$ and $\:{\text{M}\text{R}}_{\text{p}\text{r}\text{e},\:\text{i}}$are the i^th^ experimental and predicted values; $\:{\stackrel{-}{\text{M}}\text{R}}_{\text{p}\text{r}\text{e}}\:$is the average predicted values; N is the number of observations; n is the constants number in a model.

## Results and discussions

During the drying tests, the ambient air temperature ranged from 25 to 32 °C (in winter) and from 36 to 38 °C (in summer).

### Effect of drying temperature, air change rate, and drying season on weight loss of basil

**Fig. 7:**
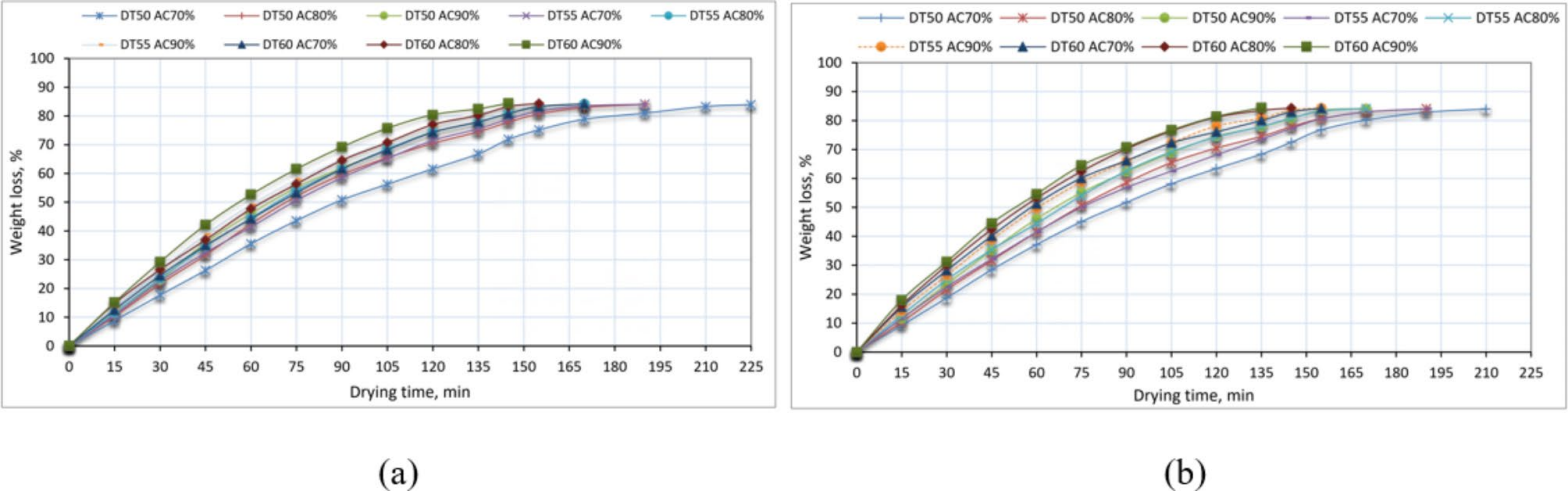
Effect of drying temperature, air change rate, and drying season on weight loss of basil, whereas (**a**) winter season, and (**b**) summer season.

Some agricultural products lose a lot of weight, which hurts their quality and makes them less profitable. For example, they might lose shape or texture, or the color might turn bad^75^. The primary cause of weight loss is the process of leaching and diffusion when water-soluble elements such as vitamins, tastes, minerals, carbohydrates, sugars, and proteins are released from plant tissue into the surroundings^76^. Figure 7 shows the weight loss of basil samples due to changes in drying temperature, air change rate, and drying season. The illustrated data in the same figure showed that the weight loss of basil did not differ for drying sessions, while the weight loss of basil was increased with increasing the air temperature, where the highest weight loss of basil was observed at an air temperature of 60 °C and the lowest weight loss of basil was observed at an air temperature of 50 °C, meaning that the weight loss increases with increasing power, the higher the power, the more weight loss. Kidmose and Kaack^77^ and Wang et al.^75^ also experienced a comparable phenomenon.

As anticipated, the early stages of the drying process saw a higher weight loss, and as the drying time increased, the MC significantly decreased, losing more than 60% of the weight of the basil sample in the first 90 min. These results agree with Singh and Gaur^34^ and Khater et al.^16^. Also, we found that weight loss increases with increasing air changing rate, where the relative humidity of the new air suctioned from outside the DR is less than the hot air inside the DR, where the drying process led to the evaporation of free water from the dried product into the surrounding environment, which increased the relative humidity inside the DR. In this case, increasing the air change rate means losing more energy, which leads to burning more PLG to maintain the air temperature inside the DR. Furthermore, Fig. 7a and b show that summer drying was faster than winter drying because of the relative humidity and ambient temperature variances. Summer drying took 135–210 min, and winter drying took 145–225 min to reach equilibrium MC.

### Effect of drying temperature, air change rate, and drying season on drying rate of basil

**Fig. 8:**
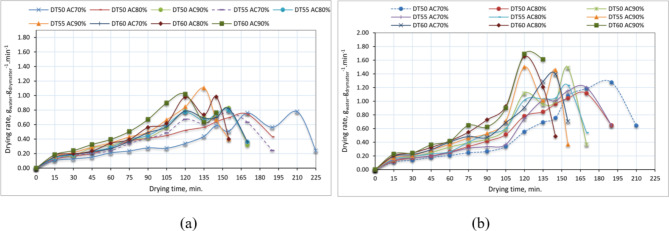
Effect of drying temperature, air change rate, and drying season on drying rate of basil, whereas (**a**) winter season, and (**b**) summer season.

Figure 8 displays the drying rate curves of purple basil leaves across various drying temperatures, air change rates, and drying seasons. The drying rates showed a general decline with time and as the MC dropped. According to the curves shown in Fig. 8, a consistent drying rate was observed in the drying process of basil leaves, specifically during the decreasing rate period. The process of drying commenced immediately. The presence of bound water facilitates the internal diffusion process, leading to the falling-rate period.

The drying process begins with the extraction of water from the larger capillaries, then progresses to the smaller capillaries, resulting in a reduction in the rate of evaporation^78^. The final stage of the process involves extracting water that is tightly bonded to water-holding components such as protein and starch. This makes the extraction process more challenging and causes the drying rate to decrease as the drying time progresses^35^.

Additionally, the highest drying rate occurred in the summer session due to the higher ambient air temperature than the minter session. Conversely, the highest moisture rate happens at 120–140 min. This is because the sun irradiation is greater after 1.0 pm on the best performance days. As previously described by Slam et al.^79^, the moisture rate increases as the drying temperature and air change rate rise.

### Effect of drying temperature, air change rate, and drying season on MR, drying coefficient, and determination coefficient of basil

**Fig. 9:**
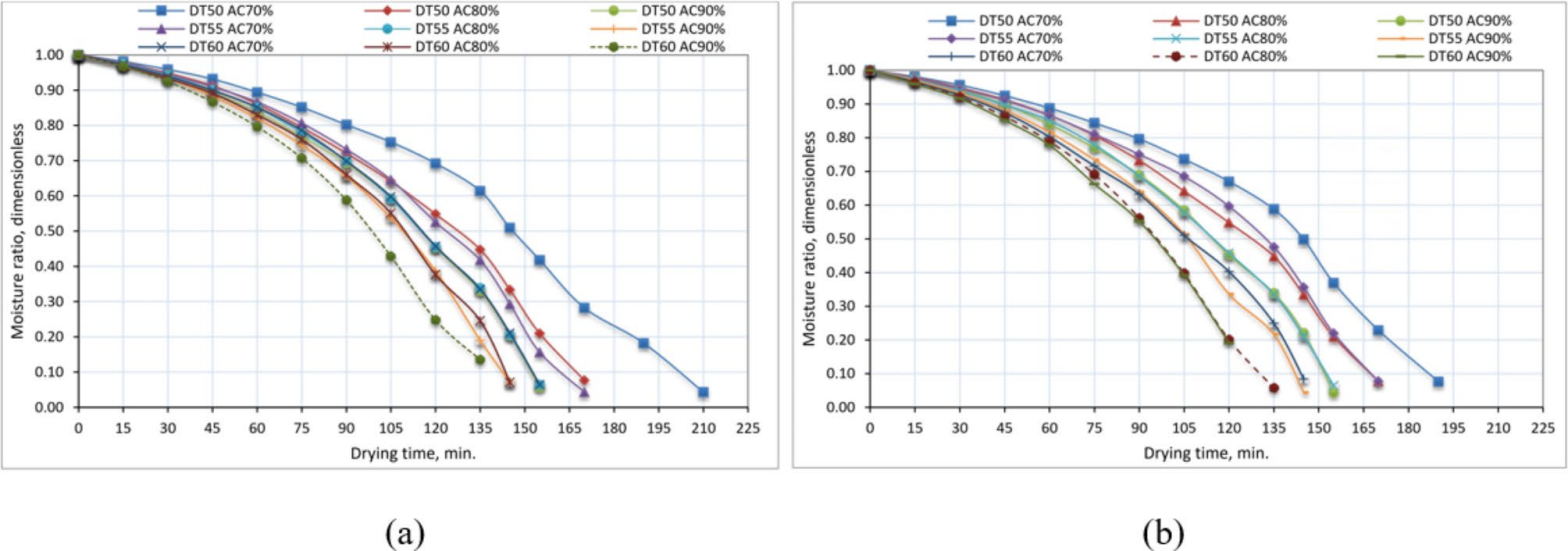
Effect of drying temperature, air change rate, and drying season on MR of basil, whereas (**a**) winter season, and (**b**) summer season.

Figure 9 displays the MR vs. drying time curves of basil for various drying temperatures, air change rates, and drying seasons. The basil leaves were subjected to drying trials until they reached a state of equilibrium MC. Figure 9 demonstrates that raising the air temperature from 50 to 60 °C leads to a reduction in the time required for the finished product to dry. This finding aligns with the outcomes reported by Beigi^80^ and Kaleta et al.^81^. During the drying process, raising the air temperature from 50 to 60 °C resulted in enhanced mass transfer, decreased process duration, and lower energy usage^82^.

According to Kara and Doymaz’s^82^ report, an increase in air temperature resulted in a reduction in the drying time for apple pomace. Beigi^80^ observed that as the air temperature within the measured range rose, there was a corresponding rise in the quantity of moisture extracted from the product. Furthermore, we observed a drop in the MR curve as the hot air temperature increased, which aligns with the findings of Sharabiani et al.^83^.

Table 2 lists the drying coefficient (k) and coefficient of determination (R^2^) for dried basil, which were determined at different drying temperatures, air change rates, and dry seasons. Compared to the drying temperatures, we did not observe an ideal trend for both coefficients, but we can say that the drying coefficient increases with an increase in the air temperature within both drying sessions. In addition, as the air change rate increased, the drying coefficient increased with an increase in the air change rate. These findings come in agreement with^81,84,85^.

**Table 2:** Drying coefficient and determination coefficient of basil drying.

Coefficient	Season	DT50°C			DT55°C			DT60°C		
		AC70%	AC80%	AC90%	AC70%	AC80%	AC90%	AC70%	AC80%	AC90%
k	W	0.011	0.011	0.013	0.014	0.013	0.014	0.013	0.014	0.013
	S	0.01	0.011	0.014	0.011	0.013	0.016	0.013	0.017	0.012
R^2^	W	0.6859	0.722	0.6874	0.6784	0.6984	0.7151	0.6956	0.6963	0.8066
	S	0.6821	0.7204	0.6469	0.6872	0.7009	0.6711	0.73	0.7264	0.7979

### Effective moisture diffusivity (EMD) and activation energy

**Fig. 10:**
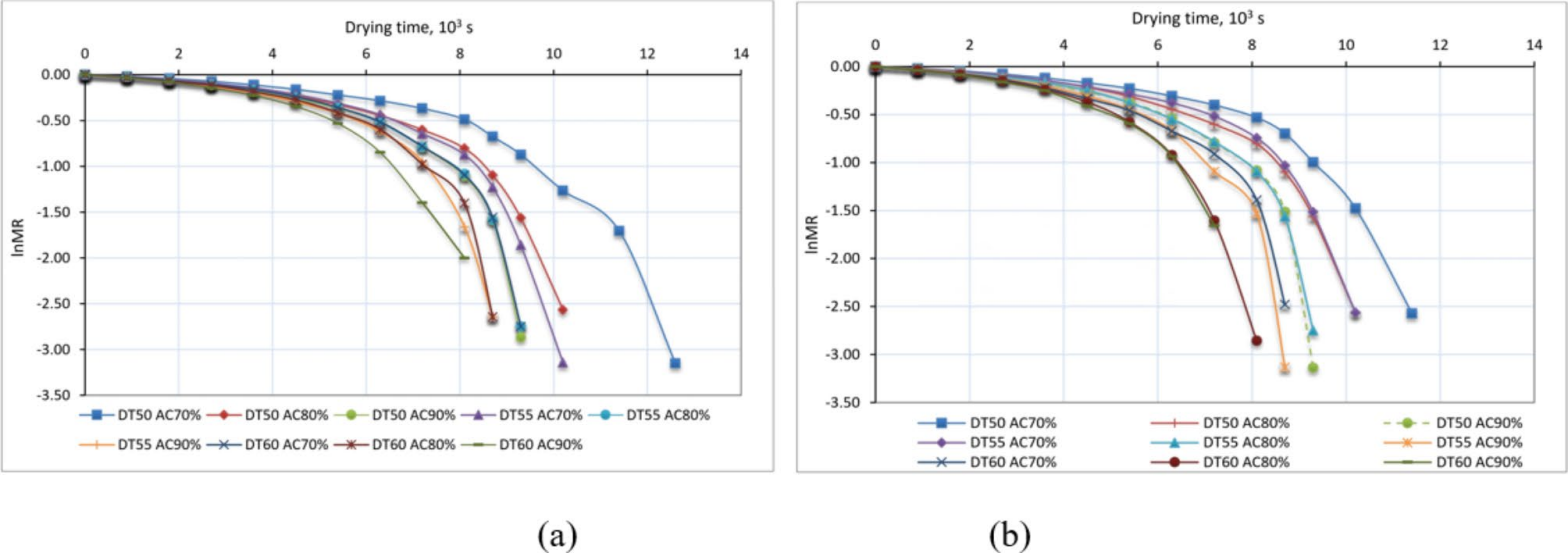
Relation between lnMR and drying time of basil drying, whereas (a) winter season, and (b) summer season.

**Fig. 11:**
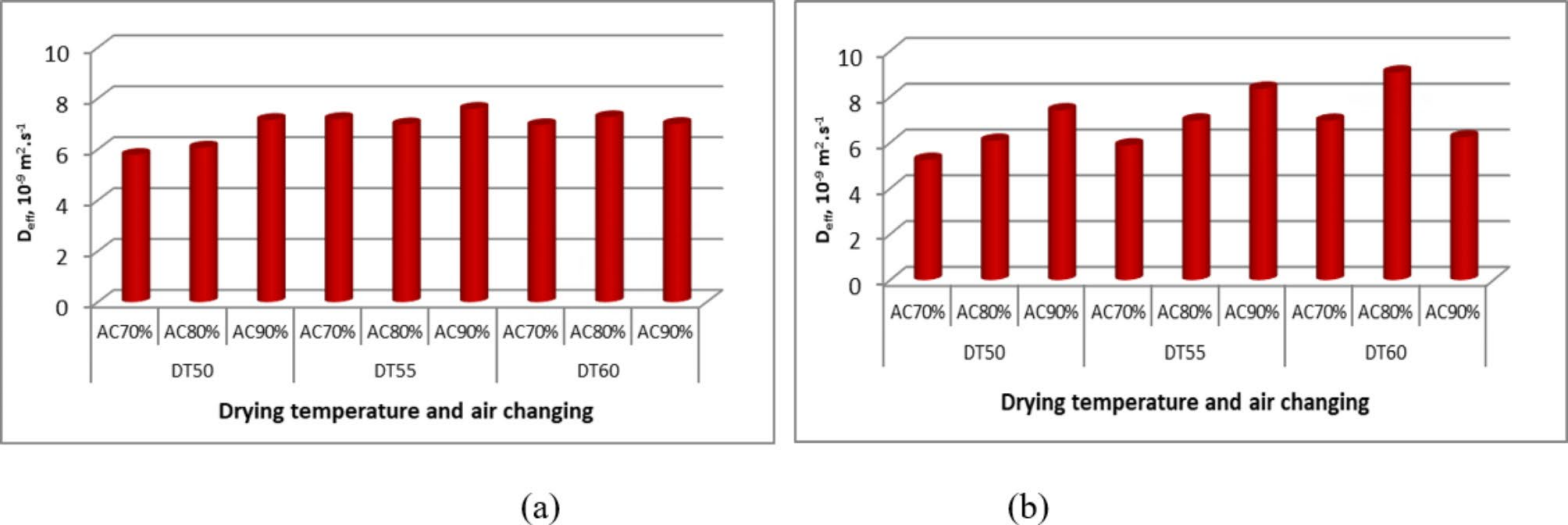
Effect of drying temperature, air change rate, and drying season on EMD of basil, whereas (a) winter season, and (b) summer season.

**Fig. 12:**
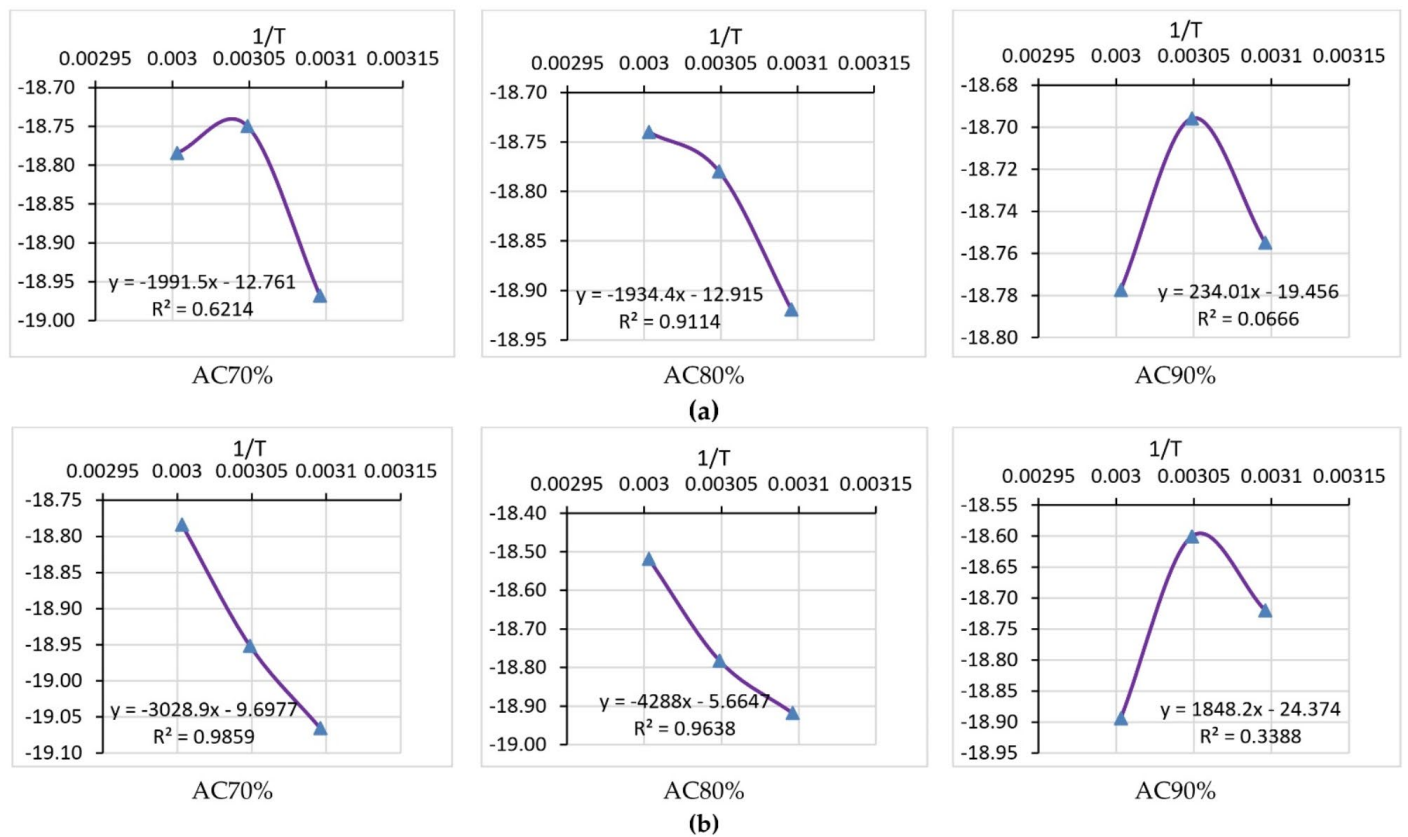
lnMR vs. drying time for different drying parameters, whereas (a) winter season, and (b) summer season.

The EMD for basil leaves was determined by applying Fick’s second law of diffusion (Eqs. 6–9) to the data obtained from the plot of the natural logarithm of MR (LnMR) against time, as shown in Figs. 10, 11 and 12. The calculated values of EMD for different temperatures, air change rates, and drying sessions are summarized in Table 3. The curves were linearly fitted with R^2^ values, indicating that liquid diffusion governs the drying process. The values of EMS exhibited a positive correlation with both the elevation of drying temperatures and the augmentation of air change rates. As depicted in Fig. 11, the EMD of basil leaves exhibited minor variations during both drying cycles. In addition, the EMD was elevated as a result of the rising drying temperature and air exchange rate. Table 3 presents a comparison of the Earth Mover’s Distance (EMD) determined in the current study with that of earlier investigations.

**Fig. 13:**
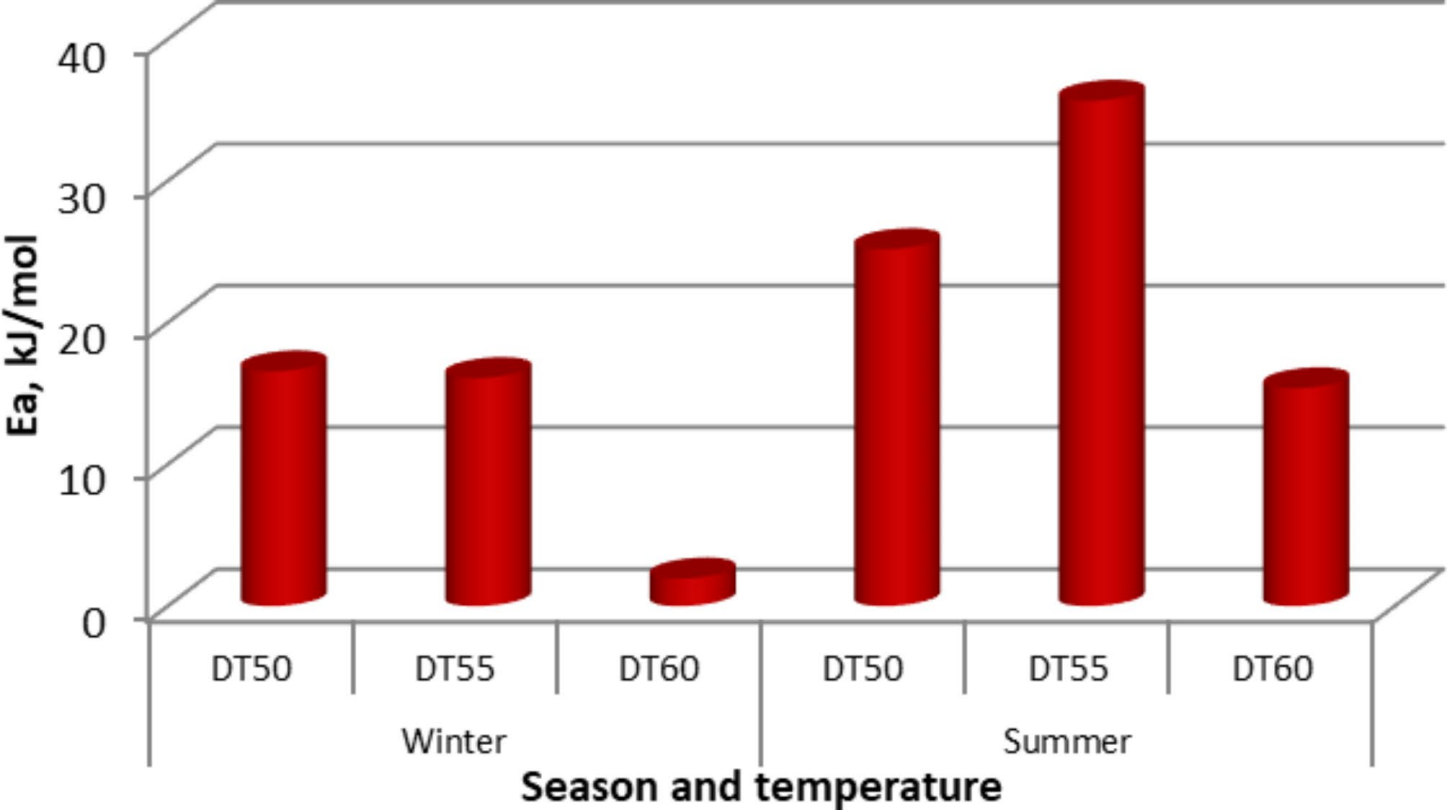
Activation energy at different drying temperatures, air changing, and drying seasons.

Figure 13 shows the activation energy of the samples, where it was calculated to find the minimum energy at which the drying occurs. For the two drying sessions (summer and winter) and three air temperatures (50, 55, and 60 °C), the activation energy decreased from 16.557 to 25.182 kJ/mol to 1.945–15.366 kJ/mol due to increasing the air temperature from 50 °C to 60 °C for the winter and summer sessions, respectively, which demonstrates that the activation energy for the summer session was higher than the activation energy for the winter session. These results are very close to those found by Shahi et al.^86^, who used solar and vacuum dryers to dry basil leaves at air temperatures of 45, 55, and 65 °C and reported that the activation energy ranged from 38.54 to 20.32 kJ/mol. Martins et al.^87^ Stated that the activation energy of basil is 39.63 kJ/mol. Mbegbu et al.^88^ studied the effect of different air temperatures on the activation energy (30–70 °C) of lemon basil leaves using a vacuum oven dryer. They stated that the activation energy was 32.34 kJ/mol. Table 3 compares the current study’s obtained EMD with previous studies.

**Table 3:** Comparison between the EMD obtained in the current study with previous studies.

Reference	Drying system	Dried product	EMD, m^2^/s
Ambawat et al.^48^	Fluidized Bed Dryer	Moringa leaves	3.59 to 2.92 × 10^− 10^
Seyedabadi^89^	Microwave	Basil leaves	1.624 to 7.652 × 10^− 10^
López-Ortiz et al.^10^	Solar greenhouses	Basil leaves	0.08 to 8.11 × 10^− 10^
Mbegbu et al.^88^	Vacuum oven dryer	Scent leaves	4.76 to 1.74 × 10^− 12^
Mbegbu et al.^88^	Vacuum oven dryer	Lemon basil leaves	4.80 to 2.06 × 10^− 12^
Altay et al.^35^	Microwave	Purple basil	0.162 to 7.09 × 10^− 8^
Current study	LPG Hybrid Solar Dryer	Basil leaves	5.25 to 9.06 × 10^− 9^

### Thin-layer kinetics

**Fig. 14:**
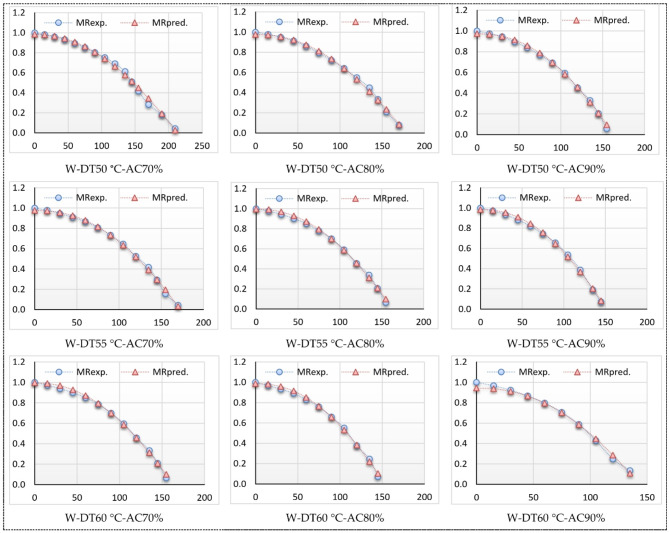
Modified Midilli II experimental and predicted MR vs. Dt (winter season).

**Fig. 15:**
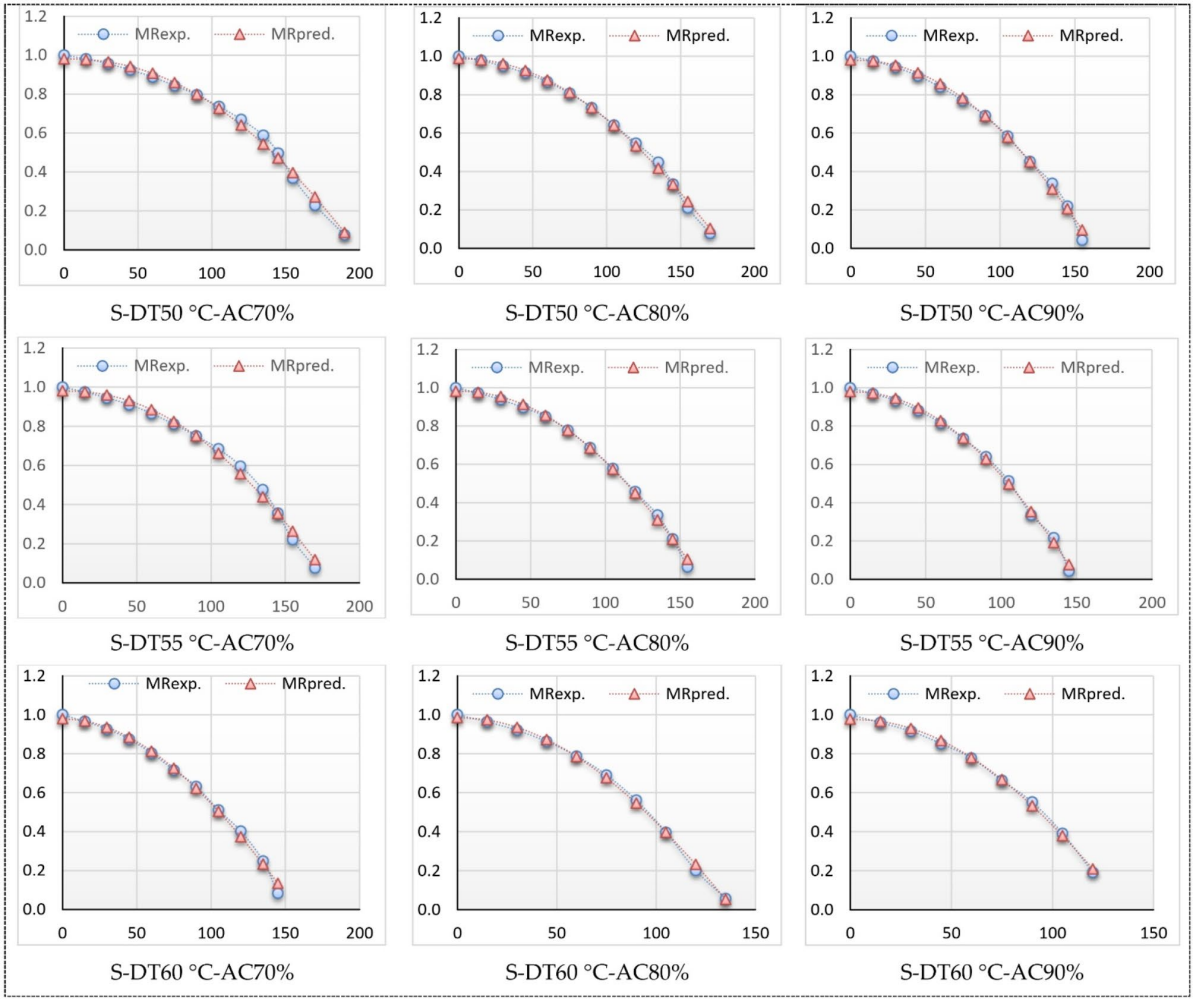
Modified Midilli II experimental and predicted MR vs. Dt (summer season).

The relationship between the MR and drying time of basil is illustrated in Figs. 14 and 15. Also, Table 4 displays the drying constants and the values of coefficient of determination (R²), reduced chi-square (χ²), and root mean square error (RMSE) for the eleven models. The MC data collected during the drying experiment under various conditions were analyzed using the 11 standard thin-layer drying models enumerated in Table 1. The statistical outcomes of several models, including the R², χ², and RMSE values, are encapsulated in Table 4. The R^2^ values for the Modified Two Term III, Newton (Lewis), and Approximation diffusion models were the lowest, ranging from 0.750512 to 0.822356, while all other models exhibited R^2^ values exceeding 0.822356. The corresponding χ^2^ and RMSE values were below 0.025451 and 0.156099, respectively. In contrast, the R^2^ values for the Modified Midilli (II) model surpassed 0.9980468, with corresponding χ^2^ and RMSE values lower than 0.0004053 and 0.015005, respectively, indicating a strong fit of the data to the Modified Midilli (II) model.

Furthermore, the Modified Midilli (II) model demonstrated superior performance in accurately fitting the experimental data obtained from the ThL hybrid LPG solar drying process throughout both winter and summer seasons, as illustrated in Figs. 14 and 15. Hence, this model was chosen to depict the drying characteristics of a thin-layer HSD during both winter and summer seasons. As illustrated in Table 4 of Modified Midilli (II) model, the highest R^2^ values were observed at air changing rate of 80% and air temperature of 55 °C, and the lowest R^2^ values were observed at 70% and air temperature of 50 °C, in winter season, while the highest R^2^ values were observed at air changing rate of 70% and air temperature of 55 °C, and the lowest R^2^ values were observed at 80% and air temperature of 50 °C, in summer season.

**Table 4:** ThL kinetics constants and statistical analysis results.

Mode No.	DT, °C	AC, %	Winter				Summer			
			Model constants	Statistical measures			Model constants	Statistical measures		
				R^2^	χ^2^	RMSE		R^2^	χ^2^	RMSE
Newton (Lewis)	50	70	k = 0.004820	0.759947	0.024846	0.151894	k = 0.004775	0.750512	0.023528	0.14737
		80	k = 0.005811	0.785106	0.022350	0.143134	k = 0.005771	0.779170	0.023116	0.145565
		90	k = 0.006465	0.776384	0.025348	0.151800	k = 0.006458	0.775545	0.025451	0.152108
	55	70	k = 0.006093	0.761717	0.027733	0.159442	k = 0.005486	0.760593	0.024132	0.148730
		80	k = 0.006372	0.771776	0.025769	0.153056	k = 0.006425	0.778244	0.024847	0.150295
		90	k = 0.006858	0.772421	0.026409	0.154169	k = 0.007117	0.777134	0.026810	0.155335
	60	70	k = 0.006344	0.769968	0.025850	0.153297	k = 0.006874	0.808348	0.020404	0.135511
		80	k = 0.006640	0.770640	0.025653	0.151946	k = 0.007870	0.782187	0.027413	0.156099
		90	k = 0.007233	0.802926	0.021473	0.138155	k = 0.007049	0.822356	0.016446	0.119958
Page	50	70	k = 1.422*10^−6^ *n* = 2.644486	0.980733	0.002230	0.043719	k = 1.422*10^−6^ *n* = 2.644486	0.9653772	0.0038175	0.056835
		80	k = 2.363*10^−6^ *n* = 2.628663	0.980289	0.002349	0.044240	k = 1.354*10^−6^ *n* = 2.7431276	0.982047	0.002152	0.042348
		90	k = 2.591*10^−6^ *n* = 2.6607618	0.978338	0.002839	0.048199	k = 1.354*10^−6^ *n* = 2.7431276	0.943427	0.008223	0.082026
	55	70	k = 2.12*10^−6^ *n* = 2.666705	0.977339	0.002997	0.049972	k = 1.265*10^−6^ *n* = 2.7434841	0.971021	0.003354	0.05287
		80	k = 2.12*10^−6^ *n* = 2.666705	0.965513	0.004765	0.062442	k = 1.35*10^−6^ *n* = 2.743128	0.948097	0.007446	0.078053
		90	k = 8.47*10^−7^ *n* = 2.936746	0.979656	0.002803	0.047353	k = 1.37*10^−6^ *n* = 2.8464125	0.981312	0.0026534	0.046073
	60	70	k = 8.47*10^−7^ *n* = 2.936746	0.949900	0.006231	0.071400	k = 1.32*10^−6^ *n* = 2.841523	0.976826	0.002946	0.048543
		80	k = 8.47*10^−7^ *n* = 2.936746	0.979284	0.002717	0.046620	k = 2.6*10^−6^ *n* = 2.767222	0.985368	0.002214	0.041499
		90	k = 8.47*10^−7^ *n* = 2.936746	0.963650	0.005357	0.064551	k = 2.861*10^−6^ *n* = 2.745217	0.983935	0.001908	0.037830
Simplified Ficks Diffusion	50	70	a = 1.156466 b = 0.054421 c = 3.011389	0.818203	0.022885	0.134094	a = 1.143276 b = 0.053805 c = 3.011536	0.807896	0.022480	0.131500
		80	a = 1.147174 b = 0.064597 c = 3.012207	0.838659	0.021197	0.126085	a = 1.148771 b = 0.064325 c = 3.012418	0.833826	0.021980	0.128393
		90	a = 1.149057 b = 0.071696 c = 3.013660	0.829363	0.025047	0.134967	a = 1.149322 b = 0.071647 c = 3.013580	0.828714	0.025151	0.135247
	55	70	a = 1.158547 b = 0.068062 c = 3.013149	0.817114	0.026849	0.141904	a = 1.141877 b = 0.061089 c = 3.011698	0.814136	0.023694	0.133307
		80	a = 1.150353 b = 0.070906 c = 3.013558	0.826231	0.025424	0.135980	a = 1.147747 b = 0.071203 c = 3.013364	0.831004	0.024526	0.133555
		90	a = 1.143753 b = 0.075842 c = 3.014551	0.824615	0.027237	0.138080	a = 1.146688 b = 0.067782 c = 2.800475	0.828669	0.027547	0.138864
	60	70	a = 1.148661 b = 0.070494 c = 3.013366	0.823743	0.025665	0.136623	a = 1.130523 b = 0.074875 c = 3.013378	0.854335	0.020735	0.120476
		80	a = 1.140572 b = 0.073494 c = 3.014100	0.823154	0.026503	0.136206	a = 1.142374 b = 0.074189 c = 2.795283	0.832089	0.029424	0.140058
		90	a = 1.131072 b = 0.079305 c = 3.014486	0.852325	0.022489	0.122446	a = 1.107367 b = 0.076365 c = 3.013739	0.866851	0.018310	0.106975
Approximation diffusion or (diffusion approach)	50	70	k = 0.004820 a = 1.000000 b = 1.000111	0.759947	0.029364	0.151894	k = 0.004775 a = 1.000000 b = 1.000131	0.750512	0.028233	0.147370
		80	k = 0.005811 a = 1.000000 b = 1.000010	0.785106	0.027316	0.143134	k = 0.005771 a = 1.000000 b = 1.000013	0.779170	0.028252	0.145565
		90	k = 0.006465 a = 1.000061 b = 1.001321	0.776384	0.031685	0.151800	k = 0.006458 a = 1.000061 b = 1.001317	0.775545	0.031813	0.152108
	55	70	k = 0.006093 a = 1.000000 b = 1.000000	0.761717	0.033896	0.159442	k = 0.005486 a = 1.000000 b = 1.000033	0.760593	0.029494	0.148730
		80	k = 0.006372 a = 1.000000 b = 1.000000	0.771776	0.032211	0.153056	k = 0.006425 a = 1.000053 b = 1.001299	0.778244	0.031059	0.150295
		90	k = 0.006858 a = 1.000000 b = 1.000000	0.772421	0.033954	0.154169	k = 0.007117 a = 1.000061 b = 1.001475	0.777134	0.034470	0.155335
	60	70	k = 0.006344 a = 1.000000 b = 1.000000	0.769968	0.032312	0.153297	k = 0.006874 a = 1.000056 b = 1.001420	0.808348	0.026233	0.135511
		80	k = 0.006640 a = 1.000000 b = 1.000000	0.770640	0.032982	0.151946	k = 0.007870 a = 1.000064 b = 1.001580	0.782187	0.036550	0.156099
		90	k = 0.007233 a = 1.000000 b = 1.000000	0.802926	0.028630	0.138155	k = 0.007049 a = 1.000000 b = 1.000039	0.822356	0.023024	0.119958
Logistics	50	70	k = 0.033442 a = 0.007640 b = 0.969197	0.991521	0.001061	0.028868	k = 0.036435 a = 0.005695 b = 0.960531	0.985612	0.001676	0.035902
		80	k = 0.035040 a = 0.013496 b = 0.982386	0.986081	0.001818	0.036923	k = 0.035907 a = 0.011890 b = 0.980226	0.987452	0.001649	0.035172
		90	k = 0.040052 a = 0.010796 b = 0.975343	0.985189	0.002159	0.039626	k = 0.039477 a = 0.011625 b = 0.978449	0.982776	0.002512	0.042741
	55	70	k = 0.040187 a = 0.007528 b = 0.968121	0.986661	0.001946	0.038200	k = 0.039057 a = 0.006608 b = 0.959718	0.731680	0.002244	0.041021
		80	k = 0.040423 a = 0.010111 b = 0.976532	0.986997	0.001889	0.037062	k = 0.039485 a = 0.011556 b = 0.978562	0.987220	0.001841	0.036592
		90	k = 0.044236 a = 0.009190 b = 0.969460	0.986086	0.002145	0.038749	k = 0.043878 a = 0.010618 b = 0.974450	0.986675	0.002125	0.038569
	60	70	k = 0.040947 a = 0.009316 b = 0.973070	0.987353	0.001828	0.036464	k = 0.037374 a = 0.020512 b = 0.990272	0.985227	0.002087	0.038226
		80	k = 0.043626 a = 0.009268 b = 0.971512	0.987014	0.001931	0.036766	k = 0.048696 a = 0.010292 b = 0.973504	0.991280	0.001514	0.031768
		90	k = 0.042794 a = 0.016095 b = 0.988960	0.995630	0.000659	0.020956	k = 0.041600 a = 0.021734 b = 0.996643	0.992521	0.001020	0.025245
Parpbolic model	50	70	a = 0.981797 b = 0.000997 c= -0.000028	0.982126	0.0023011	0.0425208	a = 0.979869 b = 0.001581 c= -0.000034	0.983041	0.002040	0.039610
		80	a = 0.977638 b = 0.001174 c= -0.000039	0.989516	0.001399	0.032393	a = 0.977641 b = 0.001303 c= -0.000040	0.989915	0.001355	0.031881
		90	a = 0.975759 b = 0.001319 c= -0.000046	0.991676	0.001234	0.029956	a = 0.975759 b = 0.001322 c= -0.000046	0.990420	0.001421	0.032142
	55	70	a = 0.977644 b = 0.001475 c= -0.000042	0.989970	0.001491	0.033442	a = 0.977645 b = 0.001640 c= -0.000041	0.985438	0.0018965	0.0377141
		80	a = 0.975762 b = 0.001460 c= -0.000047	0.991993	0.001184	0.029339	a = 0.975759 b = 0.001304 c= -0.000046	0.880399	0.001158	0.029025
		90	a = 0.974182 b = 0.001296 c= -0.000052	0.993525	0.001011	0.026603	a = 0.974178 b = 0.001089 c= -0.000052	0.905738	0.001056	0.027190
	60	70	a = 0.975762 b = 0.001494 c= -0.000047	0.991940	0.001187	0.029377	a = 0.974164 b = 0.000520 c= -0.000045	0.992205	0.001116	0.027954
		80	a = 0.974184 b = 0.001403 c= -0.000052	0.9925713	0.0011214	0.028018	a = 0.972225 b = 0.000777 c= -0.000058	0.994919	0.000886	0.024310
		90	a = 0.972222 b = 0.000604 c= -0.000053	0.993055	0.001056	0.026529	a = 0.969012 b = 0.000748 c= -0.000061	0.995288	0.0006474	0.0201157
Combined Two-term and Page	50	70	k = 1.77*10^−8^ a = 0.900427 b = 0.055797 h = 0 *n* = 3.502453	0.991026	0.001370	0.029681	k = 1.77*10^−8^ a = 0.900427 b = 0.0557966 h = 4.88*10^−5^*n* = 3.502453	0.9776039	0.0032027	0.0443948
		80	k = 1.475*10^−7^ a = 0.163326 b = 1.766724 h = 0 *n* = 3.184704	0.985795	0.002383	0.037280	k = 5.51*10^−7^ a = 0.978345 b = 0 h = 0.828593 *n* = 2.919836	0.985629	0.002445	0.037763
		90	k = 9.73*10^−8^ a = 0.1556636 b = 1.8112558 h = 0 *n* = 3.3288717	0.9851286	0.0028868	0.0396814	k = 7.73*10^−7^ a = 0.96963 b = 4.84*10^–13^ h = 0.8281 *n* = 2.90146	0.981040	0.003696	0.044898
	55	70	k = 8.15 *10^−9^ a = 0.931316 b = 0.071104 h = 0.076051 *n* = 3.784720	0.990139	0.001846	0.032816	k = 7.73*10^−7^ a = 0.96963 b = 4.84*10^–13^ h = 0.8281 *n* = 2.90146	0.903229	0.015166	0.094056
		80	k = 8.01*10^−8^ a = 0.153611 b = 1.827049 h = 0 *n* = 3.366660	0.9870612	0.0025029	0.0369492	k = 1.42*10^−5^ a = 0.471051 b = 0.740586 h = 2.93*10^−7^ *n* = 2.365	0.898142	0.018825	0.101333
		90	k = 1.23*10^−5^ a = 0.477917 b = 0.736921 h = 2.55*10^−8^ *n* = 2.364999	0.969856	0.006551	0.057232	k = 1.42*10^−5^ a = 0.471051 b = 0.740586 h = 2.93*10^−7^ *n* = 2.36500	0.963810	0.007847	0.062639
	60	70	k = 5.95*10^−8^ a = 0.139154 b = 1.923194 h = 0 *n* = 3.4260879	0.987419	0.002422	0.036348	k = 1.42*10^−5^ a = 0.471051 b = 0.740586 h = 2.93*10^−5^ *n* = 2.36500	0.959504	0.007704	0.062063
		80	k = 1.189*10^−5^ a = 0.4764031 b = 0.7416107 h = 1.683*10^−7^ *n* = 2.365005	0.9703076	0.0062155	0.0557474	k = 1.585*10^−5^ a = 0.472046 b = 0.748449 h = 5.72*10^−7^ *n* = 2.369975	0.977314	0.005910	0.051249
		90	k = 1.42*10^−5^ a = 0.471051 b = 0.740586 h = 2.93*10^−7^ *n* = 2.364996	0.9861212	0.0031528	0.0374334	k = 1.51 *10^−5^ a = 0.468468 b = 0.7413035 h = 8.153*10^−7^*n* = 2.3678759	0.985940	0.003191	0.034590
Modified Henderson and Pabis	50	70	k = 0.006001 a = 0.385488 b = 0.385488 c = 0.385488 g = 0.006001 h = 0.006001	0.818205	0.031467	0.134094	k = 0.0059330 a = 0.381094 b = 0.381094 c = 0.381094 g = 0.005933 h = 0.005933	0.807896	0.032114	0.131500
		80	k = 0.007119 a = 0.382393 b = 0.382393 c = 0.382393 g = 0.007119 h = 0.007119	0.838661	0.031795	0.126085	k = 0.007089 a = 0.382925 b = 0.382925 c = 0.382925 g = 0.007089 h = 0.007089	0.833826	0.032969	0.128393
		90	k = 0.007894 a = 0.383021 b = 0.383021 c = 0.383021 g = 0.007894 h = 0.007894	0.829365	0.040075	0.134967	k = 0.007889 a = 0.383108 b = 0.383108 c = 0.383108 g = 0.007889 h = 0.007889	0.828714	0.040242	0.135247
	55	70	k = 0.007497 a = 0.386186 b = 0.386186 c = 0.386186 g = 0.007497 h = 0.007497	0.817114	0.040274	0.141904	k = 0.006735 a = 0.380627 b = 0.380627 c = 0.380627 g = 0.006735 h = 0.006735	0.814135	0.035541	0.133307
		80	k = 0.007808 a = 0.383451 b = 0.383451 c = 0.383451 g = 0.007808 h = 0.007808	0.826233	0.040679	0.135980	k = 0.007841 a = 0.382584 b = 0.382584 c = 0.382584 g = 0.007841 h = 0.007841	0.831005	0.039241	0.133555
		90	k = 0.008346 a = 0.381253 b = 0.381253 c = 0.381253 g = 0.008346 h = 0.008346	0.824617	0.047665	0.138080	k = 0.008643 a = 0.382232 b = 0.382232 c = 0.382232 g = 0.008643 h = 0.008643	0.828669	0.048208	0.138864
	60	70	k = 0.007763 a = 0.382889 b = 0.382889 c = 0.382889 g = 0.007763 h = 0.007763	0.823743	0.041065	0.136623	k = 0.008246 a = 0.376843 b = 0.376843 c = 0.376843 g = 0.008246 h = 0.008246	0.854334	0.036286	0.120476
		80	k = 0.008090 a = 0.380190 b = 0.380190 c = 0.380190 g = 0.008090 h = 0.008090	0.823156	0.046380	0.136206	k = 0.009495 a = 0.380794 b = 0.380794 c = 0.380794 g = 0.0094950 h = 0.009495	0.832089	0.058849	0.140058
		90	k = 0.008727 a = 0.377019 b = 0.377019 c = 0.377019 g = 0.008727 h = 0.008727	0.852324	0.044979	0.122446	k = 0.008408 a = 0.369124 b = 0.369124 c = 0.369124 g = 0.008408 h = 0.008408	0.866851	0.045775	0.106975
**Modified Midilli II**	**50**	**70**	k = 3.3 *10 ^**−6**^ **a = 4.443862** **b= -3.45960** *n* = 2.09567	**0.994123**	**0.000809**	**0.024032**	**k = 1.66E-06** **a = 5.06309** **b= -4.08148** *n* = 2.22364	**0.993452**	**0.000845**	**0.024187**
		**80**	k = 3.38*10 ^**−6**^ **a = 4.282044** **b= -3.30477** *n* = 2.169711	**0.997028**	**0.000435**	**0.017029**	**k = 5.25E-06** **a = 4.48591** **b=-3.49774** *n* = 2.07230	**0.996951**	**0.000451**	**0.017343**
		**90**	k = 2.49*10 ^**−6**^ **a = 4.539418** **b= -3.56713** *n* = 2.25330	**0.996794**	**0.000534**	**0.018438**	k = 3.63*10 ^**−6**^ **a = 5.00884** **b=-4.02813** *n* = 2.15849	**0.995558**	**0.0007400**	**0.021703**
	**55**	**70**	k = 2.03*10 ^**−6**^ **a = 4.64283** **b= -3.66614** *n* = 2.26392	**0.997264**	**0.000447**	**0.017264**	k = 2.62*10 ^**−6**^ **a = 4.62358** **b=-3.64277** *n* = 2.19499	**0.992446**	**0.001077**	**0.026798**
		**80**	k = 3.91*10 ^**−6**^ **a = 4.45665** **b=-3.46075** *n* = 2.17337	**0.996473**	**0.000590**	**0.019381**	k = 4.61*10 ^**−6**^ **a = 4.77000** **b=-3.78691** *n* = 2.12085	**0.997374**	**0.000432**	**0.016580**
		**90**	k = 3.73*10 ^**−6**^ **a = 4.45003** **b=-3.46611** *n* = 2.21326	**0.997087**	**0.000524**	**0.017739**	k = 5.76*10 ^**−6**^ **a = 5.00204** **b=-4.02157** *n* = 2.10017	**0.997144**	**0.000530**	**0.017831**
	**60**	**70**	k = 3.90*10 ^**−6**^ **a = 4.45636** **b=-3.46074** *n* = 2.17308	**0.996403**	**0.000599**	**0.019520**	k = 1.44*10 ^**−5**^ **a = 4.20683** **b=-3.22727** *n* = 1.93945	**0.995399**	**0.000756**	**0.021299**
		**80**	k = 3.57*10 ^**−6**^ **a = 4.44847** **b=-3.46125** *n* = 2.21818	**0.996202**	**0.000662**	**0.019924**	k = 1.237*10 ^**−5**^ **a = 4.319012** **b = 3.333210** *n* = 2.015851	**0.9980468**	**0.0004053**	**0.015005**
		**90**	k = 3.51*10 ^**−6**^ **a = 4.42572** **b=-3.48175** *n* = 2.24171	**0.992372**	**0.001358**	**0.027465**	k = 1.11*10 ^**−5**^ **a = 3.53175** **b=-2.55400** *n* = 2.08978	**0.997275**	**0.000464**	**0.015237**
Modified Page III	50	70	k = 1.15648 d = 2.80675 *n* = 0.04728	0.818204	0.022885	0.134094	k = 1.14329 d = 2.82822 *n* = 0.04745	0.807896	0.022480	0.131500
		80	k = 1.14718 d = 2.80233 *n* = 0.05591	0.837325	0.021197	0.126085	k = 1.14878 d = 2.80324 *n* = 0.05570	0.833826	0.021980	0.128393
		90	k = 1.14906 d = 2.79585 *n* = 0.06171	0.829364	0.025047	0.134967	k = 1.14932 d = 2.79581 *n* = 0.06167	0.828715	0.025151	0.135247
	55	70	k = 1.15855 d = 2.79427 *n* = 0.05853	0.817115	0.026849	0.141904	k = 1.14188 d = 2.81244 *n* = 0.05327	0.814136	0.023694	0.133307
		80	k = 1.15036 d = 2.79691 *n* = 0.06108	0.826232	0.025424	0.135980	k = 1.14775 d = 2.79671 *n* = 0.06133	0.831005	0.024526	0.133555
		90	k = 1.14376 d = 2.86202 *n* = 0.06836	0.824615	0.027237	0.138080	k = 1.14670 d = 2.85105 *n* = 0.07025	0.828669	0.027547	0.138864
	60	70	k = 1.14867 d = 2.79798 *n* = 0.06078	0.823744	0.025665	0.136623	k = 1.13052 d = 2.85919 *n* = 0.06741	0.854334	0.020735	0.120476
		80	k = 1.14058 d = 2.86839 *n* = 0.06656	0.823153	0.026503	0.136206	k = 1.14238 d = 2.78691 *n* = 0.07375	0.832090	0.029424	0.140058
		90	k = 1.131075 d = 2.812327 *n* = 0.069026	0.852324	0.022489	0.122446	k = 1.10737 d = 2.79496 *n* = 0.06568	0.866851	0.018310	0.106975
Modified Two Term III	50	70	k = 0.004820 a = 1.000001	0.759947	0.026917	0.151894	k = 0.00478 a = 1.00000	0.750512	0.025667	0.147370
		80	k = 0.005811 a = 1.000000	0.785106	0.024585	0.143134	k = 0.00577 a = 1.00000	0.779170	0.025427	0.145565
		90	k = 0.006465 a = 1.000005	0.776384	0.028164	0.151800	k = 0.00646 a = 1.00000	0.775545	0.028278	0.152108
	55	70	k = 0.00609 a = 1.00001	0.761717	0.030506	0.159442	k = 0.00549 a = 1.00000	0.760593	0.026545	0.148730
		80	k = 0.006372 a = 1.000004	0.771776	0.028632	0.153056	k = 0.00643 a = 1.00000	0.778244	0.027608	0.150295
		90	k = 0.00686 a = 1.00000	0.772421	0.029710	0.154169	k = 0.00712 a = 1.00001	0.7771339	0.0301613	0.1553352
	60	70	k = 0.00634 a = 1.00000	0.769968	0.028722	0.153297	k = 0.00687 a = 1.00000	0.808348	0.022954	0.135511
		80	k = 0.00664 a = 1.00000	0.770640	0.028860	0.151946	k = 0.00787 a = 1.00001	0.782187	0.031329	0.156099
		90	k = 0.00723 a = 1.00000	0.802926	0.024540	0.138155	k = 0.007049 a = 1.000000	0.822356	0.019187	0.119958

Figures 14 and 15 depict the relationship between predicated and experimental MC values versus drying time. The data predicted by the Modified Midilli (II) model, used for the ThL hybrid solar drying process, shows that the drying seasons often follow a straight line. This indicates that the chosen models accurately represent the drying properties of basil leaves. This results were come in agreement with the presented data in Ref. [95].

## Conclusion and future works

All solar dryers worldwide face significant challenges due to the fluctuation of air temperature and relative humidity within the drying room (DR), prompting researchers to explore methods to manage this issue. Therefore, the current study aimed to design an automatic control system to control both air temperature and relative humidity inside the drying room and regulate the drying process using two different energy sources (LPG and solar energy). Then, the developed automatic LPG hybrid solar dryer was used for drying basil leaves at three different air temperatures of 50, 55, and 60 °C, with there air changing rates of 70, 80, and 90%, and the drying process was repeated in two seasons of summer and winter. The obtained results showed that the weight loss of basil did not significantly affect for both drying seasons, but it increased with air temperature. Also, the drying time in summer was shorter than winter by about 35.71–35.56%. A consistent drying rate was observed during the decreasing rate period. Additionally, the values of effective moisture diffusivity ranged from 5.25 to 9.06 × 10^− 9^ m^2^/s, which exhibited a positive correlation with both the elevation of drying temperatures and air-changing rates. The analysis of activation energy revealed a direct correlation with air drying temperature, with the winter season exhibiting the highest activation energy. On the other hand, the thin layer modeling of the drying process showed that the Modified Midilli (II) model exhibits the highest R^2^ and the lowest χ^2^ and RMSE values for both winter and summer seasons. It also demonstrated superior performance in accurately fitting the experimental data.

## Potential future works

In the current study, the primary focus was the examination of various variables associated with the dried product only. Consequently, additional experiments can be undertaken to appraise the effectiveness of the existing dryer in terms of energy, exergy, environmental impact, and economic viability.

## Data Availability

All data are presented within the article.

## Abbreviations

LPG: Liquid petroleum gas
HSD: Hybrid solar dryer
IoT: Internet of things
OSD: Open sun drying
SD: Solar dryers
DR: Drying room
SAH: Solar air heater
MC: Moisture content
ThL: Thin layer
EMD: Effective moisture diffusivity
EA: Economic analysis
MR: Moisture ratio
FPSC: Flat plate solar collector
WL: Flat plate solar collector (FPSC)
RMSE: Root means square error
